# Responses of different invasive and non-invasive ornamental plants to water stress during seed germination and vegetative growth

**DOI:** 10.1038/s41598-023-40517-7

**Published:** 2023-08-16

**Authors:** Diana M. Mircea, Roberta Calone, Elena Estrelles, Pilar Soriano, Radu E. Sestras, Monica Boscaiu, Adriana F. Sestras, Oscar Vicente

**Affiliations:** 1 Department of Forestry, University of Agricultural Sciences and Veterinary Medicine Cluj-Napoca, 3-5 Manastur Street, 400372 Cluj-Napoca, Romania; 2 CREA-Council for Agricultural Research and Economics, Research Centre for Agriculture and Environment, 40128 Bologna, Italy; 3 CREA-Council for Agricultural Research and Economics, Research Centre for Agriculture and Environment, 00184 Rome, Italy; 4 Cavanilles Institute of Biodiversity and Evolutionary Biology, Botanical Garden, University of Valencia, Quart, 80, 46008 Valencia, Spain; 5 Department of Horticulture and Landscape, University of Agricultural Sciences and Veterinary Medicine Cluj-Napoca, 3-5 Manastur Street, 400372 Cluj-Napoca, Romania; 6 Mediterranean Agroforestry Institute (IAM), Universitat Politècnica de València, Camino de Vera s/n, 46022 Valencia, Spain; 7 Institute for the Conservation and Improvement of Valencian Agrodiversity (COMAV), Universitat Politècnica de València, Camino de Vera s/n, 46022 Valencia, Spain

**Keywords:** Ecology, Plant sciences

## Abstract

Biological invasions represent a major threat to natural ecosystems. A primary source of invasive plants is ornamental horticulture, which selects traits related to invasiveness. This study evaluated the responses to water stress during germination and vegetative growth of six species used as ornamental or medicinal plants. Three of them are recognised as invasive weeds in many world areas. Seeds were exposed to increasing concentrations of polyethylene glycol (PEG) mimicking drought stress, and young plants in the vegetative growth stage were subjected to two levels of water stress. Results indicated that in the absence of stress in control conditions, the most competitive species were those reported as weeds, namely *Bidens pilosa* L*.*, *Oenothera biennis* L*.*, and *Centaurea cyanus* L*.*, the last regarding germination velocity. Under stress, only two species, *Limonium sinuatum* (L.) Mill. and *C. cyanus*, maintained germination at –1 MPa osmotic potential, but in the recovery experiment, an osmopriming effect of PEG was observed. The most tolerant species during growth were two natives in the Mediterranean region, *L. sinuatum* and *Lobularia maritima* (L.) Desv., both accumulating the highest proline concentrations. The sixth species studied, *Echinacea purpurea* (L.) Moench., proved to be more susceptible to stress in the two developmental stages. This study reveals that the most significant traits associated with invasiveness were related to germination, especially in the absence of stress.

## Introduction

Biological invasions represent one of the main threats to biodiversity worldwide, favoured by globalisation and climate change. The economic importance of plants is a driver of their potential invasiveness, ornamental, medicinal, culinary or fodder species having the highest likelihood of naturalisation^1^. The primary source of invasive plants is ornamental horticulture, as many were intentionally introduced for ornamental purposes but also accidentally as seed contaminants, transported with other cargo, or in ballast soil^2,3^. Some qualities desirable in ornamentals are also attributes of invasive plants, such as the capacity for environmental adaptation, rapid germination and prolific seedling emergence, rapid vegetative growth, early flowering, or prolonged flowering periods^3^. Therefore, ornamentals may accidentally favour plant invasions by selecting genotypes with spread-promoting traits.

Climate change will boost plant invasions globally, but its consequences could vary by region^4^. As species respond differently to environmental changes, some, like increased CO_2_, may promote invasion, while others, like temperature and precipitation, may prevent it^5^. Ornamental species from warmer climes can expand into temperate zones when temperatures rise^4^. Global warming will provide such species with a “head start” in naturalising outside their native ranges before other species migrate due to changing climates^6^. In addition, stress-tolerant ornamentals are already favoured in some areas in anticipation of changes triggered by climate change^7^. Another trait of invasive plants is their increased phenotypic plasticity, which generally gives them a greater fitness advantage^8^. The significance of climate change's effects on biological invasions is reflected in an increasing number of studies on invasive species' responses to abiotic stress under different experimental conditions^9–11^ and comparative studies of functional traits in invasive and native species^2,12,13^. However, a binary classification into invasive and non-invasive ornamentals is problematic since invasiveness is not discrete, and species can be native to one place but invasive to another^14^. Furthermore, when it comes to ornamental plants, cultivated but not yet naturalised species may become future invaders if climates become more favourable to their spread^15^. Climate change makes gardens “jumping-off points” for invasion^6^.

In the present study, we evaluated the performance under water stress of six species included in the Global Compendium of Weeds^16,17^ with different invasiveness risk scores. Three of these (*B. pilosa*, *C. cyanus* and *O. biennis*), although cultivated in some areas as ornamental or medicinal plants, are agricultural and environmental weeds classified with a high-risk score; two are ornamental (*L. sinuatum* and *L. maritima*) with a medium risk score; and one (*E. purpurea*), cultivated as an ornamental and medicinal plant, although reported in some areas as naturalised, has not yet been scored^17^. The six species are spread across many world regions as cultivated or naturalised but have different biogeographical origins. They are not taxonomically closely related, with three belonging to different tribes of the large family Asteraceae and the rest to other dicot families.

Selecting different materials had several goals: (i) to identify traits associated with invasiveness under optimal (control) experimental conditions; (ii) to assess whether these species behave similarly under water stress conditions during germination and early vegetative growth; and (iii) to reveal their basic biochemical mechanisms underlying their growth responses to stress, such as photosynthetic pigment degradation, osmolyte synthesis, and antioxidant activation. This is the first comparative study of the responses to osmotic stress during germination and to water scarcity during vegetative growth in these six species and the first relating their tolerance to stress to their different invasive potential. In addition, some of the species have never before been analysed from a biochemical point of view.

## Results

### Germination and seedling analysis

**Figure 1:**
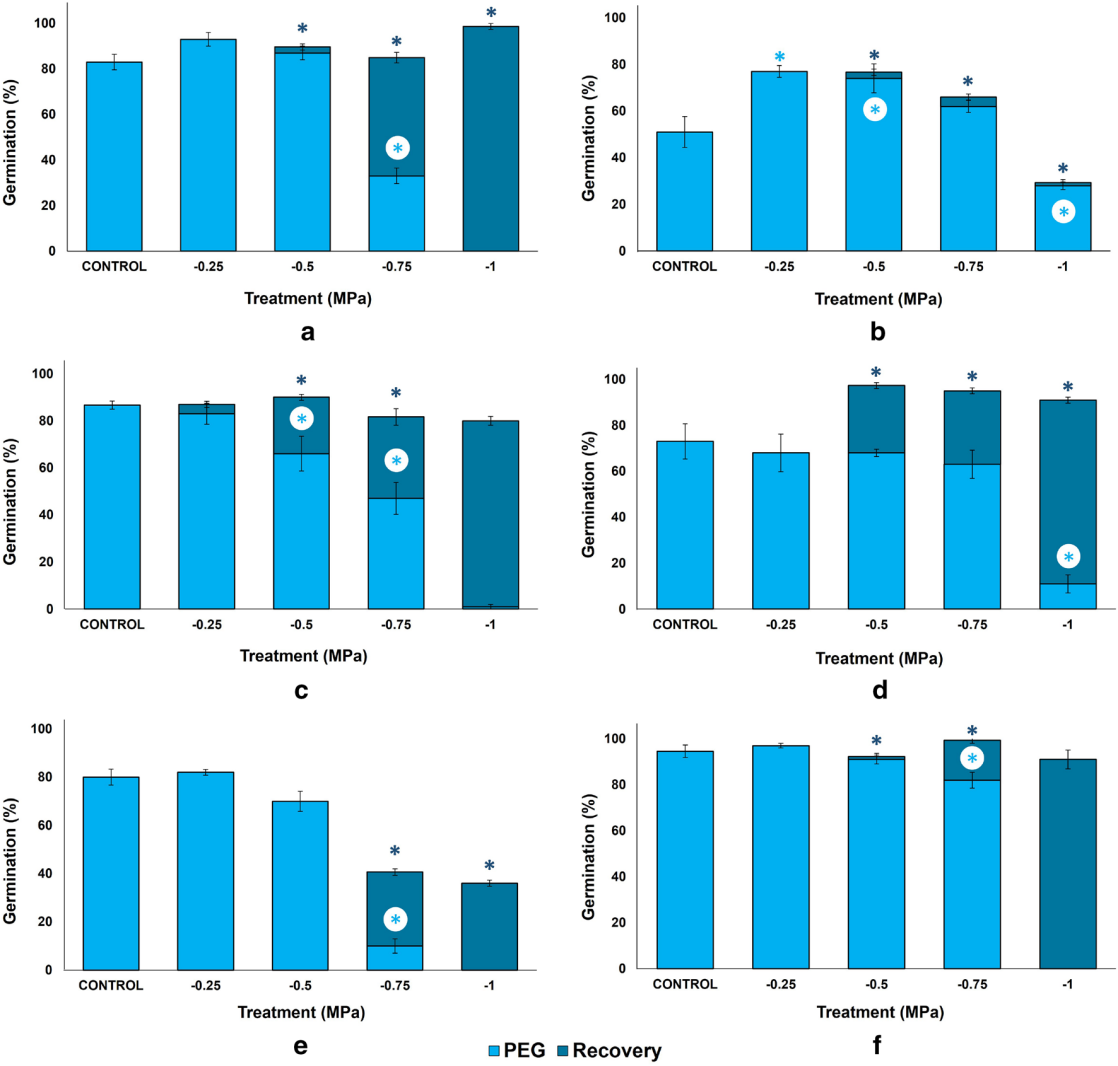
Final germination percentages after 30 days under increasing concentrations of PEG (light blue) and germination recovery percentages after 20 days in distilled water (dark blue) for (**a**) *Bidens pilosa*, (**b**) *Centaurea cyanus*, (**c**) *Echinacea purpurea,* (**d**) *Limonium sinuatum,* (**e**) *Lobularia maritima,* (**f**) *Oenothera biennis*. Control: germination in distilled water. The values shown are means ± SEM (n = 4). Asterisks indicate significant differences in each condition with respect to the corresponding control (according to Dunnett test, p < 0.05).

Under control conditions, without stress, all species had germination percentages above 80%, except *C. cyanus* with only 51% and *L. sinuatum* with 73%. The highest germination, 94.6%, was found in *O. biennis* (Fig. 1).

There were no significant differences in final germination percentages between treatments at the lowest PEG concentration, except for *C. cyanus*, whose germination was stimulated. At − 0.50 MPa the only significant reduction was found *E. purpurea* and an increase in *C. cyanus*. Germination was reduced in the − 0.75 MPa PEG treatment in all species except *C. cyanus*, where it was higher but not statistically different than in the absence of osmotic stress. The strongest reduction was observed in *L. maritima* and the smallest in *L. sinuatum* and *O. biennis*. Under the highest concentration of PEG, only seeds of *C. cyanus* and *L. sinuatum* germinated. Summarising, *C. cyanus* proved to be the most tolerant to osmotic stress, followed by *L. sinuatum*. In *O. biennis,* seed germination was wholly inhibited at − 1 MPa, but it had the highest germination percentage at − 0.75 MPa (Fig. 1).

Seeds that did not germinate in the presence of PEG had good recovery capacity when placed in new Petri dishes with distilled water for 20 days (Fig. 1). Complete recovery was observed in *B. pilosa*, *E. purpurea*, *L. sinuatum* and *O. biennis*, with final germination percentages close to controls. In *B. pilosa* and *L. sinuatum*, seed recovery treatment under − 1 MPa stress resulted in 98.7% and 80% final germination percentages, significantly higher than their respective controls. The evolution of germination during the 30 days of the experiment is shown in Supplementary Fig. 1.

**Figure 2:**
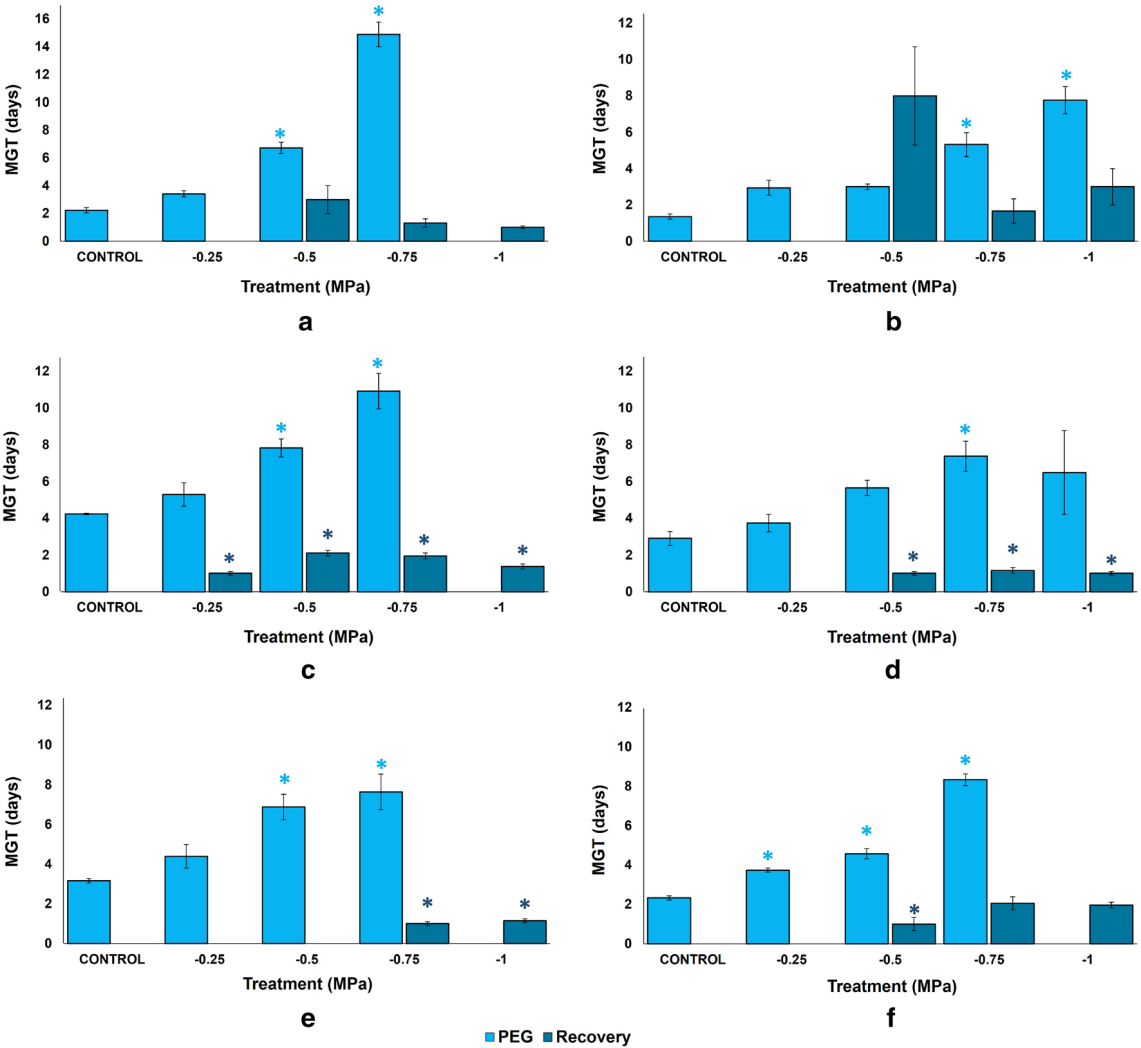
Mean Germination Time (MGT) of seeds of the six studied species after 30 days of the indicated PEG treatments (light blue) and after 20 days of “recovery” in distilled water (dark blue), in (**a**) *Bidens pilosa*, (**b**) *Centaurea cyanus*, (**c**) *Echinacea purpurea,* (**d**) *Limonium sinuatum,* (**e**) *Lobularia maritima,* (**f**) *Oenothera biennis*. Control: germination in distilled water. The values shown are means ± SEM (n = 4). Asterisks indicate significant differences in each condition with respect to the corresponding control (according to Dunnett test, p < 0.05).

Mean germination time (MGT) was calculated during stress and the subsequent recovery assays (Fig. 2). In the absence of stress, the fastest germination was registered in *C. cyanus* (MGT = 1.4), *B. pilosa* (2.2), and *O. biennis* (2.3). The first two started germination after one day. TSG—total spread of germination—was shortest in *C. cyanus*, *O. biennis*, and *B. pilosa* and longest in *L. sinuatum* and *E. purpurea* (Suppl. Table S1). PEG concentrations gradually increased germination time. *Bidens pilosa* and *E. purpurea* seeds had the highest MGT at − 0.75 MPa. The shortest MGTs in the presence of PEG were registered in *C. cyanus*, *L. maritima* and *O. biennis*. In the recovery assay (Fig. 2), all stressed seeds that remained ungerminated had very short MGTs, except *C. cyanus*. In *E. purpurea*, *L. sinuatum* and *L. maritima* germination was faster in recovery than in control in all seeds.

Other germination variables reflecting the differences between the studied species (reduction of germination percentage; first day of germination; last day of germination; spread of germination time; germination speed; germination index) are detailed in Table S1 of the Supplementary Materials.

The study of seedlings revealed significant differences in the size of the radicle and hypocotyl between the species studied (Table 1). *Bidens pilosa* and *C. cyanus* had the longest overall length, followed by *L. sinuatum*, *E. purpurea*, *L. maritima*, and *O. biennis*. The seedling vigour index (SVI) had a higher value in *B. pilosa* (24.6) than in the others, with values ranging from 10 to 15. PEG caused an overall progressive decrease in seedling length in all species. Radicle length in *L. maritima*, *L. sinuatum*, and *B. pilosa* did not change appreciably up to − 0.5 MPa. The hypocotyl size of seedlings was considerably smaller at − 0.75 MPa, with *E. purpurea* suffering the greatest effect. In both the radicle and the hypocotyl of the two species germinating at − 1 MPa, *C. cyanus* showed a lesser reduction than *L. sinuatum*.

**Table 1:** Seedling's parameters (means ± SEM; n = 4) calculated for the six studied species in the 30-day germination assays.

Parameter	MPa PEG	*B. pilosa*	*C. cyanus*	*E. purpurea*	*L. sinuatum*	*L. maritima*	*O. biennis*
Radicle length (mm)	0	32.7 ± 1.7 c (0%)	23.9 ± 1.7 d (0%)	11.4 ± 0.2 e (0%)	16.1 ± 1.5 b (0%)	8.0 ± 0.3 b (0%)	11.3 ± 0.2 d (0%)
	−0.25	23.7 ± 0.6 b (27.5%)	18.9 ± 0.6 c (21.0%)	7.2 ± 0.1 d (36.1%)	16.0 ± 1.0 b (0.4%)	9.1 ± 0.1 b (−12.8%)	8.1 ± 0.0 c (28.4%)
	−0.5	20.7 ± 0.5 b (36.6%)	13.0 ± 0.7 b (45.4%)	4.4 ± 0.1 c (61.5%)	12.1 ± 0.7 b (24.7%)	8.6 ± 0.2 b (−7.5%)	7.5 ± 0.0 b (33.9%)
	−0.75	8.7 ± 1.0 a (73.3%)	9.7 ± 0.4 b (59.1%)	2.1 ± 0.0 b (81.6%)	4.1 ± 0.2 a (74.1%)	3.7 ± 1.2 a (54.2%)	3.3 ± 0.1 a (70.4%)
	−1	n.g	4.1 ± 0.1 a (82.7%)	n.g	1.3 ± 0.4 a (91.5%)	n.g	n.g
Hypocotyl length (mm)	0	29.9 ± 2.8 b (0%)	28.1 ± 0.3 e (0%)	11.9 ± 0.2 d (0%)	20.4 ± 1.8 c (0%)	16.9 ± 0.5 c (0%)	12.0 ± 0.4 d (0%)
	−0.25	25.6 ± 0.3 b (14.3%)	19.8 ± 0.8 d (29.5%)	8.2 ± 0.0 c (31.5%)	16.6 ± 0.7 c (18.4%)	12.8 ± 0.1 b (24.2%)	6.9 ± 0.1 c (41.9%)
	−0.5	24.4 ± 0.3 b (18.5%)	11.1 ± 0.5 c (60.3%)	5.3 ± 0.1 b (55.8%)	10.3 ± 0.4 b (49.3%)	11.8 ± 0.2 b (30.3%)	4.5 ± 0.1 b (62.7%)
	−0.75	8.6 ± 0.3 a (71.1%)	8.6 ± 0.2 b (69.2%)	0 a (100%)	5.4 ± 0.2 a (73.6%)	4.3 ± 1.4 a (74.7%)	2.8 ± 0.0 a (76%)
	−1	n.g	5.7 ± 0.3 a (79.5%)	n.g	1.6 ± 0.5 a (91.8%)	n.g	n.g
SVI	0	24.6 ± 1.6 b	14.3 ± 1.7 c	10.3 ± 0.2 d	15.2 ± 2.8 d	13.6 ± 1.0 c	11.4 ± 0.6 d
	−0.25	23.8 ± 0.6 b	15.2 ± 0.2 c	6.8 ± 0.3 c	11.2 ± 1.2 cd	10.5 ± 0.1 b	6.8 ± 0.1 c
	−0.5	21.2 ± 0.5 b	8.2 ± 0.5 b	3.5 ± 0.4 b	7.0 ± 0.4 bc	8.3 ± 0.5 b	4.1 ± 0.1 b
	−0.75	2.8 ± 0.2 a	5.3 ± 0.1 b	0 a	3.4 ± 0.4 ab	0.5 ± 0.2 a	2.4 ± 0.1 a
	−1	n.g	1.6 ± 0.1 a	n.g	0.2 ± 0.1 a	n.g	n.g

Different lowercase letters indicate significant differences between treatments per species, according to the Tukey test (p < 0.05).

*n.g.* no germination.

Percentages of reduction shown in parenthesis were calculated considering the mean in control as 100%.

### Plant growth analysis

As expected, there were large differences in growth parameters between the six species (Table 2). *Bidens pilosa* was the most vigorous species with the highest shoot growth rate and total fresh weight. Vigorous growth was observed in *L. sinuatum*, especially in leaves, whereas *E. purpurea* had the greatest development of roots in proportion to leaves. *L. maritima* showed a large increase in shoot length, but due to its specific morphology with numerous, small, and narrow leaves, it had the lowest total fresh weight.

**Table 2:** Growth parameters measured after 30 days of stress treatments (CON—control, IWS—intermediate water stress and SWS—severe water stress) in the six ornamental species.

Parameter	Treat	*B. pilosa*	*C. cyanus*	*E. purpurea*	*L. sinuatum*	*L. maritima*	*O. biennis*
Root length (cm)	C	29.2 ± 1.9 a (0%)	12.8 ± 2 a (0%)	26.2 ± 2.2 b (0%)	37.6 ± 5.7 a (0%)	28.1 ± 5 a (0%)	31.9 ± 8.1 b (0%)
	IWS	28.4 ± 1.5 a (2.6%)	13.1 ± 2.1 a (−2.4%)	21.2 ± 1.2 ab (19.1%)	40.3 ± 5.2 a (−7%)	37.3 ± 4.1 a (−32.7%)	20.9 ± 2.8 ab (34.2%)
	SWS	22.9 ± 2.8 a (21.3%)	9.4 ± 0.9 a (26.7%)	19.9 ± 1.1 a (24%)	34 ± 4.6 a (9.7%)	29.4 ± 4.1 a (−4.4%)	10.4 ± 1.7 a (67.1%)
Root fresh weight (g)	C	6.9 ± 1.4 ab (0%)	3.8 ± 1.1 b (0%)	6.3 ± 0.9 b (0%)	2.5 ± 0.5 b (0%)	0.4 ± 0.1 a (0%)	4.8 ± 0.3 b (0%)
	IWS	7.6 ± 1.1 b (−9.4%)	2.9 ± 0.5 ab (22.6%)	3.3 ± 0.9 a (47.4%)	1.1 ± 0.1 a (54.8%)	0.3 ± 0 a (16.3%)	3.9 ± 0.4 b (18.4%)
	SWS	2.6 ± 0.6 a (61.3%)	0.4 ± 0.1 a (87.7%)	0.7 ± 0.1 a (88.1%)	0.6 ± 0.1 a (73.2%)	0.3 ± 0.1 a (25.8%)	0.4 ± 0 a (91.6%)
Root water content (%)	C	66.3 ± 1.6 b	81.3 ± 1.2 c	82 ± 0.7 c	70.9 ± 2.4 b	35.3 ± 9 a	85.4 ± 0.3 b
	IWS	53.9 ± 2.2 b	59 ± 3.5 b	70.1 ± 2.7 b	61.2 ± 7.4 ab	39.1 ± 8.6 a	81.6 ± 0.7 b
	SWS	32.4 ± 5.4 a	32.6 ± 5.4 a	29.6 ± 3 a	48.1 ± 5.3 a	61.6 ± 3.5 a	31.2 ± 9.1 a
Leaf number	C	31.6 ± 3.4 b (0%)	42 ± 3.8 ab (0%)	10 ± 0.7 b (0%)	19.5 ± 2.2 a (0%)	123.5 ± 8 a (0%)	29.8 ± 1.7 c (0%)
	IWS	32.2 ± 4.1 b (−1.8%)	63.6 ± 11.2 b (−51.4%)	9 ± 0.6 ab (10%)	19.1 ± 2.2 a (1.7%)	125.5 ± 14.4 a (-1.6%)	23.2 ± 0.9 b (14.4%)
	SWS	13.8 ± 1.8 a (56.3%)	23.2 ± 4.5 a (44.7%)	6.8 ± 0.6 a (32%)	18.1 ± 1.5 a (6.8%)	110.6 ± 11.4 a (10.3%)	14.4 ± 1.2 a (51.6%)
Shoot length (cm)	C	58.2 ± 2.7 b (0%)	19.8 ± 2.7 a (0%)	26.3 ± 2.2 a (0%)	25.5 ± 1.1 b (0%)	28.9 ± 2.3 a (0%)	17.6 ± 0.7 b (0%)
	IWS	50.6 ± 2 b (13%)	27 ± 2.8 a (−36.2%)	24.8 ± 2.1 a (5.5%)	24.2 ± 0.9 ab (5.1%)	26.5 ± 2.1 a (8%)	16.5 ± 0.4 b (6.6%)
	SWS	38.9 ± 2.3 a (33%)	19.3 ± 1.2 a (2.5%)	20.2 ± 1.2 a (23.1%)	20.9 ± 0.8 a (18%)	22.6 ± 1.8 a (21.7%)	10.6 ± 0.3 a (39.7%)
Shoot fresh weight (g)	C	35.3 ± 2.5 b (0%)	24.3 ± 2.8 b (0%)	13.6 ± 1.2 b (0%)	39.2 ± 8.4 b (0%)	13.6 ± 1.8 b (0%)	23.8 ± 1.9 c (0%)
	IWS	26 ± 2.8 b (26.3%)	18.6 ± 3.3 b (23.2%)	11.2 ± 1.9 ab (17.8%)	27.4 ± 1.8 ab (30.1%)	10 ± 0.9 b (26.5%)	18.4 ± 0.5 b (22.7%)
	SWS	8.7 ± 1.9 a (75.2%)	3.9 ± 1.2 a (83.6%)	3 ± 0.5 b (77.8%)	14.4 ± 2.8 a (63.2%)	4.2 ± 0.6 a (68.6%)	1.5 ± 0.1 a (93.5%)
Shoot water content (%)	C	87.5 ± 0.5 b	89 ± 0.8 b	85.3 ± 0.5 a	93.8 ± 0.6 a	89.6 ± 0.6 a	89.5 ± 2.3 b
	IWS	86.3 ± 0.2 b	81.7 ± 2.7 ab	84.6 ± 0.3 a	90.4 ± 1.7 a	85.5 ± 1.7 a	86.2 ± 0.2 b
	SWS	56.7 ± 11.3 a	57.8 ± 13.7 a	63.3 ± 11.1 a	73.8 ± 10.8 a	77.4 ± 6.2 a	73.8 ± 3.5 a

Values are means ± SEM (n = 5). Different lowercase letters indicate significant differences between treatments for each determined variable and species, according to the Tukey test (p < 0.05). Percentages of reduction shown in parenthesis were calculated considering the mean in control as 100%.

Most growth parameters were not affected by the intermediate water stress (IWS) treatment, except for a significant reduction of root fresh weight in *L. sinuatum* and *E. purpurea* and shoot weight in *O. biennis*. Only *E. purpurea* and *C. cyanus* showed a significant decrease in root water content under IWS conditions, whereas leaf number was reduced in *O. biennis*. Severe water stress (SWS) reduced the growth of all investigated species to different extents, as shown by the measured changes in growth traits. For example, the fresh weight of shoots (FWs) fell in all species, with *O. biennis* showing the greatest reduction (93.5%), followed by *C. cyanus*, *E. purpurea*, *L. sinuatum* and *L. maritima.* This latter species appeared to be relatively less affected by lack of watering since no other parameter, apart from FWs, showed a significant reduction under SWS conditions. On the contrary, all determined growth variables were significantly reduced in *O. biennis*, the species most sensitive to water deficit considering the reduction in total fresh weight, including roots, over 90% of control values. According to this criterion, *L. sinuatum* and *L. maritima* were the most resistant to severe water stress, reducing total FW by 64% and 68%, respectively (Table 2).

### Biochemical parameters

Slight differences were registered between the concentrations of photosynthetic pigments measured in the studied species, with maximum values of total chlorophylls (*a* and *b*) (16.7 mg g^−1^ DW) and carotenoids (3 mg g^−1^ DW) in *L. maritima* and minimal in *E. purpurea* (8.8 and 1 mg g^−1^ DW, respectively). The stress treatment induced a general decreasing trend in photosynthetic pigment contents, highlighted especially under SWS in *B. pilosa*, where chlorophylls and carotenoids dropped to half compared to the control (Table 3).

**Table 3:** Effect of stress treatments on the shoot contents of photosynthetic pigments, osmolytes and antioxidant compounds: chlorophylls *a* and *b* (Chl A and Chl B), carotenoids (Caro), proline (Pro), total soluble sugars (TSS), malondialdehyde (MDA), hydrogen peroxide (H_2_O_2_), total phenolic compounds (TPC) and total flavonoids (TF).

Biochemical analysis	Treat	*B. pilosa*		*C. cyanus*		*E. purpurea*		*L. sinuatum*		*L. maritima*		*O. biennis*
Chl A (mg g^−1^ DW)	C	10.8 ± 1.9 b		7.6 ± 1.3 a		6.7 ± 0.5 a		11.2 ± 0.5 a		13.3 ± 2.4 a		10.9 ± 2.6 a
	IWS	8.7 ± 0.9 ab		5.8 ± 1.4 a		7.0 ± 0.7 a		8.2 ± 0.6 a		5.9 ± 1.5 a		6.6 ± 0.3 a
	SWS	4.9 ± 1.1 a		4.1 ± 1.1 a		5.0 ± 0.6 a		7.6 ± 1.6 a		11.1 ± 3.2 a		5.7 ± 0.9 a
Chl B (mg g^−1^ DW)	C	3.4 ± 0.8 b		2.8 ± 0.4 a		2.1 ± 0.1 a		3.4 ± 0.2 a		3.4 ± 0.7 a		3.5 ± 0.6 a
	IWS	2.9 ± 0.4 ab		1.5 ± 0.3 a		1.6 ± 0.4 a		2.4 ± 0.1 a		1.3 ± 0.3 a		2.3 ± 0.2 a
	SWS	1.8 ± 0.3 a		1.6 ± 0.3 a		1.5 ± 0.2 a		2.3 ± 0.4 a		2.8 ± 0.9 a		2.6 ± 0.2 a
Caro (mg g^−1^ DW)	C	1.6 ± 0.3 b		1.0 ± 0.1 a		1.0 ± 0.0 a		2.2 ± 0.2 b		3.0 ± 0.7 a		2.5 ± 0.5 a
	IWS	0.8 ± 0.1 ab		1.1 ± 0.3 a		1.1 ± 0.1 a		1.7 ± 0.1 b		1.1 ± 0.3 a		1.7 ± 0.1 a
	SWS	0.8 ± 0.2 a		0.6 ± 0.1 a		0.8 ± 0.0 a		0.8 ± 0.1 a		2.1 ± 0.6 a		1.9 ± 0.2 a
Pro (µmol g^−1^ DW)	C	15.5 ± 6.2 a		8.5 ± 0.9 a		3.2 ± 0.7 a		4.3 ± 0.3 a		25.1 ± 6.8 a		2.1 ± 0.4 a
	IWS	13.1 ± 5.6 a		17.0 ± 2.2 a		4.0 ± 0.9 a		21.0 ± 3.4 a		117.7 ± 29.9 b		2.3 ± 0.3 a
	SWS	85.0 ± 7.1 b		171.4 ± 23.3 b		110.7 ± 14.8 b		313.7 ± 38.2 b		253.4 ± 28 c		11.2 ± 0.9 b
TSS (mg eq. Glu g^−1^ DW)	C	18.7 ± 1.2 a		15.5 ± 1.1 a		7.1 ± 1.8 a		16.0 ± 6.6 a		9.5 ± 0.9 a		74.5 ± 22.6 a
	IWS	19.6 ± 2.9 a		18.1 ± 3.0 a		11.0 ± 1.3 a		16.7 ± 6.7 a		23.4 ± 3.7 b		46.7 ± 2.4 a
	SWS	24.1 ± 3.7 a		20.9 ± 2.1 a		15.8 ± 4.2 a		10.6 ± 2.5 a		12.8 ± 3.0 a		60.6 ± 15.6 a
MDA (nmol g^−1^ DW)	C	488.1 ± 35.1 b		698.8 ± 81.8 a		284.6 ± 40.4 a		171.4 ± 37.8 a		166.5 ± 8.6 ab		388.5 ± 114.2 a
	IWS	440.2 ± 28 b		526.0 ± 132.7 a		246.7 ± 46.4 a		127.9 ± 20.9 a		229.4 ± 33.2 b		193.6 ± 16.6 a
	SWS	221.5 ± 36 a		487.6 ± 119.7 a		221.8 ± 82.9 a		156.6 ± 52.5 a		144.9 ± 16.8 a		220.8 ± 97.4 a
H_2_O_2_ (µmol g^−1^ DW)	C	4.1 ± 0.4 ab		2.7 ± 0.5 a		1.4 ± 0.2 a		1.8 ± 0.2 ab		0.4 ± 0.0 a		14.8 ± 5.0 a
	IWS	6.4 ± 1.0 b		2.4 ± 0.1 a		1.6 ± 0.0 a		0.8 ± 0.1 a		1.3 ± 0.4 a		4.2 ± 0.6 a
	SWS	3.2 ± 0.4 a		3.0 ± 1.3 a		1.3 ± 0.2 a		5.0 ± 1.5 b		0.7 ± 0.2 a		4.6 ± 0.5 a
TF (mg eq. C g^−1^ DW)	C	10.4 ± 1 a		1.7 ± 0.2 a		4.0 ± 1.2 a		5.2 ± 1.8 a		1.6 ± 0.2 a		18.2 ± 5.6 a
	IWS	7.0 ± 1.1 a		2.9 ± 0.7 ab		11.4 ± 2.2 b		5.1 ± 1.1 a		2.7 ± 0.4 a		9.0 ± 0.1 a
	SWS	9.3 ± 2.6 a		4.4 ± 0.7 b		5.7 ± 0.7 ab		5.9 ± 1.3 a		1.8 ± 0.2 a		18.3 ± 9.4 a
TPC (mg eq. GA g^−1^ DW)	C	17.6 ± 1.5 b		14.1 ± 1.3 a		9.7 ± 1.8 a		16.3 ± 4.0 a		5.1 ± 0.2 a		26.3 ± 6.3 a
	IWS	14.3 ± 1.4 b		13.3 ± 2.5 a		17.8 ± 2.4 b		16.4 ± 3.7 a		5.7 ± 1.2 a		12.8 ± 0.4 a
	SWS	6.3 ± 0.3 a		9.8 ± 2.6 a		9.2 ± 1.5 a		19.7 ± 4.4 a		6.0 ± 0.8 a		23.9 ± 14 a

Values shown are means ± SEM (n = 5). Different lowercase letters indicate significant differences between treatments for each determined variable and species, according to the Tukey test (p < 0.05).

*Glu* glucose, *GA* gallic acid, *C* catechin.

Proline (Pro) did not vary significantly in the IWS treatment, except for a ca. 4.7-fold increase in *L. maritima*. Under SWS conditions, Pro levels increased significantly in plants of all species, albeit to different extents, from 5.3-fold in *O. biennis* to 73-fold in *L. sinuatum*. In controls, *L. maritima* and *B. pilosa* had notably higher levels of Pro than the other taxa. The lowest Pro concentrations were detected in *O. biennis* in all treatments. The water deficit treatments did not cause any significant change in total soluble sugars (TSS) contents in the investigated species. TSS concentrations in *O. biennis* were considerably higher than in the other species under all tested conditions but especially in the non-stressed controls, from 4 to 10 fold (Table 3).

The oxidative stress markers, malondialdehyde (MDA) and hydrogen peroxide (H_2_O_2_), did not show any significant increase in the IWS or SWS treatments with respect to the corresponding controls. Accordingly, only slight variations, generally non-significant, were observed in antioxidant compounds contents, total phenolic compounds (TPC) and total flavonoids (TF) (Table 3).

#### Two-way ANOVA and multivariate analysis

Two-way ANOVA revealed significant differences between species, treatments, and their interactions for all parameters related to germination (Table S2). Growth traits shown in Table S3 also largely differed between species, except reductions in shoot length, fresh weight of roots and shoots, and water content of shoots that were similar in the six species but significantly different according to the treatment. Finally, significant differences between species were found for biochemical parameters (Table S4), but only photosynthetic pigments, proline and MDA varied according to the treatment, and interaction species × treatment were significant only for proline and MDA.

#### PCAg—germination phase

**Figure 3:**
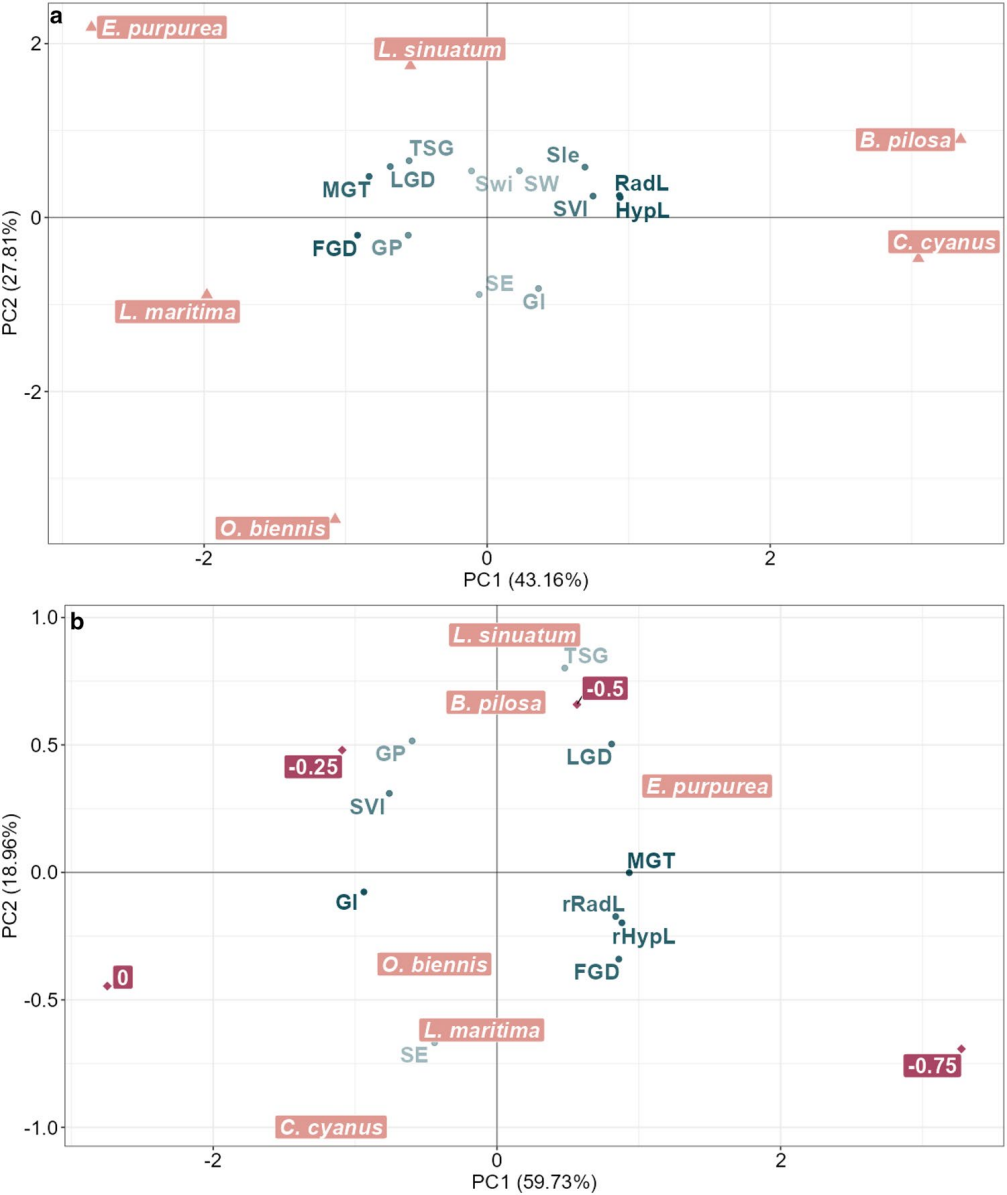
PCA biplot of the germination data under control (**a**) and drought stress (**b**). Dark-green points show the barycentre of the quantitative variables. The increasing colour intensity indicates the progressive importance of the variables in defining the first two principal components. The pink triangles indicate the barycentre of the six ornamental species. The magenta rhombus indicated the barycentre of the drought-stress treatments. The amount of variation explained by each principal component, PC1 and PC2, is indicated in parenthesis. *Sle* seed length, *Swi* seed width, *SE* seed weight, *GP* germination percentages, *MGT* mean germination time, *FGD* first germination day, *LGD* last germination day, *SE* speed of emergence, *GI* germination index, *TSG* time spread of germination, *RadL* radicle length, *HypL* hypocotyl length, *rRadL* radicle length reduction, *rHypL* hypocotyl length reduction, *SVI* seedling vigour index.

Under control conditions (Fig. 3a), the first two components explained almost 70% of the data variance. The first PC showed a positive correlation with seed length (Sle), hypocotyl length (HypL), radicle length (RadL), and seedling vigour index (SVI), positioned on the positive side of the x-axis. HypL, RadL, and SVI, in turn, resulted negatively correlated with mean germination time (MGT), first germination day (FGD), and last germination day (LGD), which collectively describe the germination duration. This suggests that as the germination time increased, the development and vigour of the seedlings decreased. PC2 exhibited a positive correlation with seed width (Swi) and weight (SW) and a negative correlation with the germination index (GI) and speed of emergency (SE). These correlations imply that species characterised by wider and heavier seeds had a lower percentage of seeds germinated in the initial days of assessment. Amongst the analysed parameters, SE, Swi, SW and GI (with light colour) contributed the least to explaining the data variability under control conditions.

Under drought stress conditions (Fig. 3b), the first two principal components accounted for approximately 70% of the data variance. The first PC clearly captures the impact of escalating drought stress, as evidenced by the control treatment positioned on the far left, the highest drought stress treatment (−0.75 MPa) situated on the far right, and the intermediate treatments distributed in between. Seed weight (SW), seed length (Sle), and seed width (Swi) were excluded from drought-stress PCA because these parameters remain unaffected by drought stress. Compared to the control condition, the barycentre of MGT shifted towards the positive side of PC1, in the same direction as the −0.75 MPa treatment. This indicates that increased drought stress prolongs the time required for germination. MGT, in turn, resulted positively correlated with the barycentres of the radicle and hypocotyl length reductions and negatively correlated with SVI, GI, GP, and speed of emergence (SE), all of which were positioned on the negative side of PC1. Notably, the barycentre of *E. purpurea*, the species that exhibited the most pronounced decline in germination performance under high drought stress conditions, was placed on the rightmost side of the x-axis. In contrast, the barycentre of *C. cyanus*, the species that displayed the least sensitivity to increasing drought stress, was located on the leftmost side of PC1.

#### PCAv—vegetative growth phase

**Figure 4:**
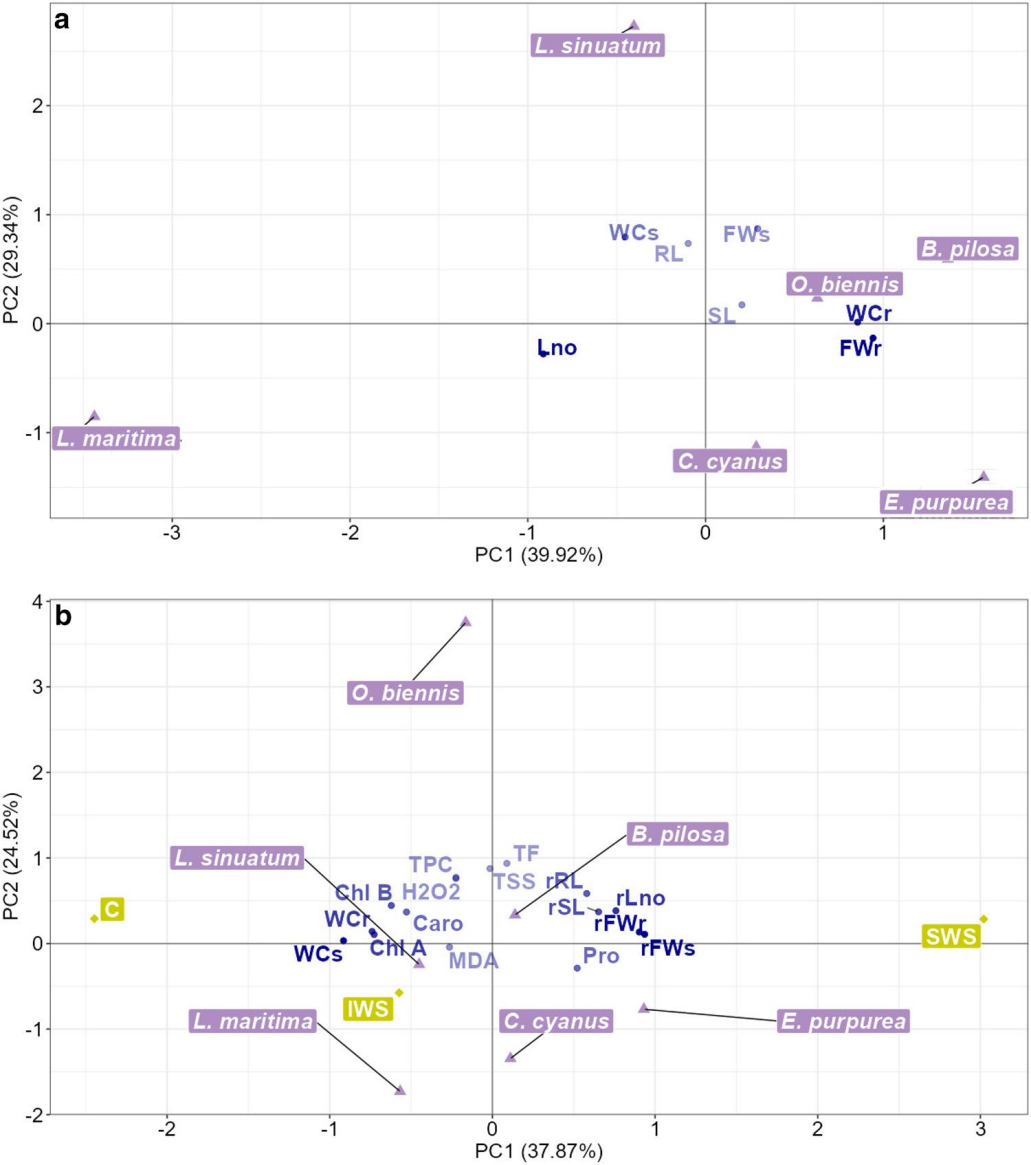
PCA biplot of the growth data under control (**a**) and drought stress (**b**). Dark-blue points show the barycentre of the quantitative variables. The increasing colour intensity indicates the progressive importance of the variables in defining the first two principal components. The violet triangles indicate the barycentre of the six ornamental species. The yellow rhombus indicated the barycentre of the drought-stress treatments. The amount of variation explained by each principal component is indicated in brackets. *RL* root length, *rRL* root length reduction, *FWr* fresh root, *rFWr* fresh weight reduction, *WCr* root water content, *SL* shoot length, *rSL* shoot length reduction, *FWr* root fresh weight, *rFWr* root fresh weight reduction, *Lno* leaf number, *rLno* leaf number reduction, *WCs* shoot water content, *Chl A* chlorophyll *a*, *Chl B* chlorophyll *b*, *Caro* carotenoids, *Pro* proline, *TSS* total soluble sugars, *MDA* malondialdehyde, *H*_*2*_*O*_*2*_ hydroxide peroxide, *TPC* total phenolics compounds, *TF* total flavonoids.

Under control conditions (Fig. 4a), the first two principal components of the PCA explained 68% of the variance in the data. A strong correlation was observed between the first PC, root fresh weight (FWr) and water content (WCr). The species *B. pilosa*, *E. purpurea*, *O. biennis*, and *C. cyanus*, located on the positive side of PC1, exhibited the highest values for FWr and WCr. In contrast*, L. maritima* and *L. sinuatum*, situated on the negative side of PC1, had smaller values of FWr and WCr. Notably, *L. maritima* stood out from all other species due to its significantly higher number of leaves. Consequently, its barycentre fell on the far left of PC1, well separated from the barycentre of the other species, and in the same direction as the barycentre of leaf number (Lno). On the other hand, the second axis showed a strong correlation with the shoot’s fresh weight and water content, namely FWs and WCs. Indeed, the barycentres of *L. sinuatum, B. pilosa,* and *O. biennis*, the species that exhibited the highest values for FWs and WCs, were located on the positive side of the y-axis. In controlled conditions, the length of the shoot and root made a less significant contribution compared to other variables in explaining the total variance of the data.

Under water stress conditions (Fig. 4b), the first two principal components explained 62% of the total data variance. PC1 clearly represents the effects of increasing water stress imposition. The control barycentre was located on the far-left side of PC1, whereas the severe water stress (SWS) barycentre was located on the far-right side of PC1. PC1 showed a strong positive correlation with the reduction in root and shoot fresh weight, as well as the content of proline. This indicates that under the highest levels of drought stress, there was a significant reduction in plant fresh weight and the highest accumulation of proline, one of the most common osmolytes produced by plants under osmotic stress. PC1 was negatively correlated with shoot and root water content, as well as the content of osmolytes. All these parameters reached their highest value under control conditions and the lowest under SWS. The second axis was strongly correlated with total soluble sugars (TSS), maximum flavonoids (TF), total phenolic compounds (TPC), and hydrogen peroxide (H_2_O_2_). This indicates that under drought stress conditions, plants experienced increased oxidative stress, leading to an overproduction of osmoregulators, such as soluble sugars, and non-enzymatic antioxidant compounds like phenols and flavonoids. *Oenothera biennis* was strongly correlated with the second axis, exhibiting the lowest values of proline but the highest values of phenols and flavonoids under drought stress. This suggests that the defence mechanism employed by this species against water stress primarily relies on phenols, flavonoids, and sugars rather than proline.

## Discussion

The results obtained indicated that the tolerance to drought differed in the developmental stages of the plants, since the same species was not the most tolerant or most susceptible during germination and vegetative growth. Different patterns were found during germination in control and under stress, with some species being more competitive in optimal conditions but not germinating at all under the lowest osmotic potential tested.

Plant invasiveness has been correlated with traits related to germination, as successful germination plays a pivotal role in the fitness of populations, plant propagation, ecological niches occupied, range of distribution, and the evolutionary potential of species^18,19^. In control, the best germination percentages were obtained in *O. biennis*, an invasive weed in many continents, including Europe. This species has a high invasive potential due to its high fecundity and ability to germinate in light, facilitating the colonisation of disturbed habitats^20^. In addition, its dormant seeds maintain their viability in the soil for a long time, up to 80 years^21^, which may also contribute to the risk of invasion. However, rapid germination is the best predictor of species' invasiveness, exceeding the percentage of germination criterion^19^. The so-called “ruderal strategy” of producing many seeds that can germinate quickly is typical for many invasive plants with high germination rates^19,22^. The fastest germination was recorded in all species in control conditions, in the absence of PEG, and it was exceptionally short in *C. cyanus*, followed by *B. pilosa*. In both, seeds started to germinate immediately after being placed into Petri dishes. Early germination means less competition from other species and earlier utilisation of resources than later germinating species, which may find it more difficult to establish. Rapid germination can also confer advantages in interspecific competition as it can hinder the establishment of neighbouring species with longer germination times^19^. Although *C. cyanus* had a reduced percentage of germination in control of only 51%, similar to previous reports^23^, due to its very short MGT and quick start of germination, it shares with *B. pilosa* traits characteristic of invasive weeds. This conclusion is further reinforced by the control PCAg, which revealed that *B. pilosa* and *C. cyanus* stood out for their notable longer hypocotyl and radicle development, and an overall higher seedling vigour index. *Echinacea purpurea*, the least prone to invasion risk of the analysed species, had, in control conditions, the highest MGT and took the longest time to finalise germination.

Climate change can significantly impact the germination patterns and phenology, which are directly related to population dynamics and the composition of communities^24^. Species with versatile germination requirements are expected to benefit from climate change^24^. Species that detect and respond more quickly to environmental changes at ecological, chorologic or genetic levels through adaptation are expected to have a selective advantage over species that respond more slowly^19,24^.

The osmotic stress induced by PEG (mimicking drought in natural habitats) strongly affected the percentage of germination only starting with − 0.75 MPa. Lower PEG concentrations did not significantly alter this parameter, except in *C. cyanus,* whose germination percentage significantly increased, and *E. purpurea,* which showed a significant decrease. At − 0.75 MPa, a significant reduction of germination was observed in *B. pilosa*, *O. biennis* and *L. maritima* and at − 1 MPa, only two species germinated, *C. cyanus* and *L. sinuatum.* The higher stress tolerance of these species is related to their ecology. The first is a weed in dry cereal fields, and the second a halophyte growing on soils with low osmotic potential; some *Limonium* species maintained maximal germination under osmotic potentials corresponding to concentrations of 200 mM NaCl^25^. On the contrary, higher PEG concentration drastically affected germination in *Lobularia maritima*, the species with the least germination at − 0.75 MPa. Although it is a species of dry Mediterranean environments, it presents temperature-activated quiescence mechanisms that prevent germination in the months before the summer drought^26^.

The germination time gradually increased with increasing PEG concentrations in all species, but mostly in *B. pilosa* and *E. purpurea,* with a calculated MGT higher than ten days at − 0.75 MPa. However, in the recovery experiment, when ungerminated seeds from each treatment were germinated in distilled water, an osmopriming effect was detected in all species except *C. cyanus,* whose seeds germinated in very high percentages under stress. Osmopriming by PEG is a popular method for alleviating the abiotic stresses in plants, as it stimulates pre-germination metabolic activities and increases the antioxidant system activities favouring radicle protrusion^27^. The highest concentration of PEG had an osmopriming effect on germination percentages in *B. pilosa* and *L. sinuatum,* and previous exposure of seeds to all concentrations reduced the time of germination in *E. purpurea*, *L. sinuatum* and *L. maritima*. A faster emergence in primed seeds was already reported in *E. purpurea*^28^.

The analysis of seedlings and SVI indicated the best performance under osmotic stress of *C. cyanus* and the worst of *E. purpurea*, where at this PEG concentration, only a small radicle was formed but not the hypocotyl. This conclusion was further supported by the stress PCAg, where the position of the barycentre for *C. cyanus* confirmed that it exhibited the highest GI and SVI values, along with the smallest reduction in seedling development under stress conditions. Additionally, the stress PCA revealed that *L. sinuatum* maintained a higher germination percentage (GP) and total spread of germination (TSG) value. *Oenothera biennis* is competitive in germination up to − 0.75 MPa, but *B. pilosa* showed a substantial reduction of its germination and an increase in MGT at this concentration. Overall, the results of the PCAg indicate that during the germination stage, *C. cyanus* exhibited the most distinct behaviour amongst all the species, showing a remarkable ability to maintain germination performance even under severe drought stress conditions, followed by *L. sinuatum*, according to their ecological requirements.

Regarding drought effects on the vegetative growth of plants, all species proved to tolerate well irrigation with half of the water used in control. In the absence of irrigation, in the SWS treatment, L. *maritima* and *L. sinuatum* stood out as the species that most effectively mitigated the harmful effects of drought stress, whereas *O. biennis* was the most susceptible one. The remaining species displayed intermediate behaviour, each exhibiting some distinguishing characteristics, albeit not as prominently as the first two, as shown by the stress PCA. For example, the position of *L. sinuatum'*s barycentre confirmed that it experienced the least reduction in plant growth under increasing water restriction, in agreement with the fact that it is a stress-tolerant Mediterranean species, as stated above. Regarding *B. pilosa*, although susceptible during germination to higher concentrations of PEG, it proved to be more tolerant during vegetative growth. Regarding environmental requirements, the species prefers warmer and more humid climates, but due to its strong tap roots, it can grow in dry places; it is also reported to withstand severe drought episodes and tolerate salinities up to 100 mM NaCl^29^.

Our findings indicate that *O. biennis* is susceptible during growth to severe water stress; therefore, its presence as invasive is more problematic in temperate and more humid regions than the Mediterranean. The species had a remarkable position in the stress PCAv, separated by all others due to its higher production of sugars, flavonoids, and phenols, even in the absence of stress, but also to the lowest Pro concentration. On the contrary, the highest Pro concentrations were reached in water-stressed plants of the two most tolerant species, *L. sinuatum* and *L. maritima*, supporting the importance of this compatible solute in drought tolerance.

## Conclusions

The most competitive species during germination in the absence of stress were *B. pilosa*, *O. biennis* and *C. cyanus*, the latter in terms of germination velocity. However, in the presence of PEG, of all analysed species, *C. cyanus* showed the best ability to tolerate osmotic stress, followed by *L. sinuatum*. *Oenothera biennis* is competitive in germination up to − 0.75 MPa, whereas *B. pilosa* showed a strong reduction of its germination and an increase in MGT at this concentration. At the highest concentration of PEG tested, none of these two species germinated. These responses were expected as *C. cyanus* is a weed typically growing in dry cereal fields, and *L. sinuatum* is a halophyte that also withstands dry climates. On the other hand, although very invasive, *O. biennis* and *B. pilosa* are restricted to areas with higher edaphic humidity. In contrast, during the vegetative phase, *L. sinuatum* and *L. maritima* stood out as the species that most effectively mitigated the negative effects of drought stress compared to the other species. The remaining species displayed intermediate behaviour, each exhibiting some distinguishing characteristics, albeit not as prominent as the two Mediterranean species. This study reveals that the most significant traits associated with invasiveness were related to germination, especially in the absence of stress. Only *L. sinuatum*, a typical halophyte, had a good performance under water stress during both developmental stages, whereas *L. maritima*, another species reported as halophyte, is tolerant to stress during vegetative growth, but its germination is affected under water deficit conditions.

## Material and methods

### Studied species

**Figure 5:**
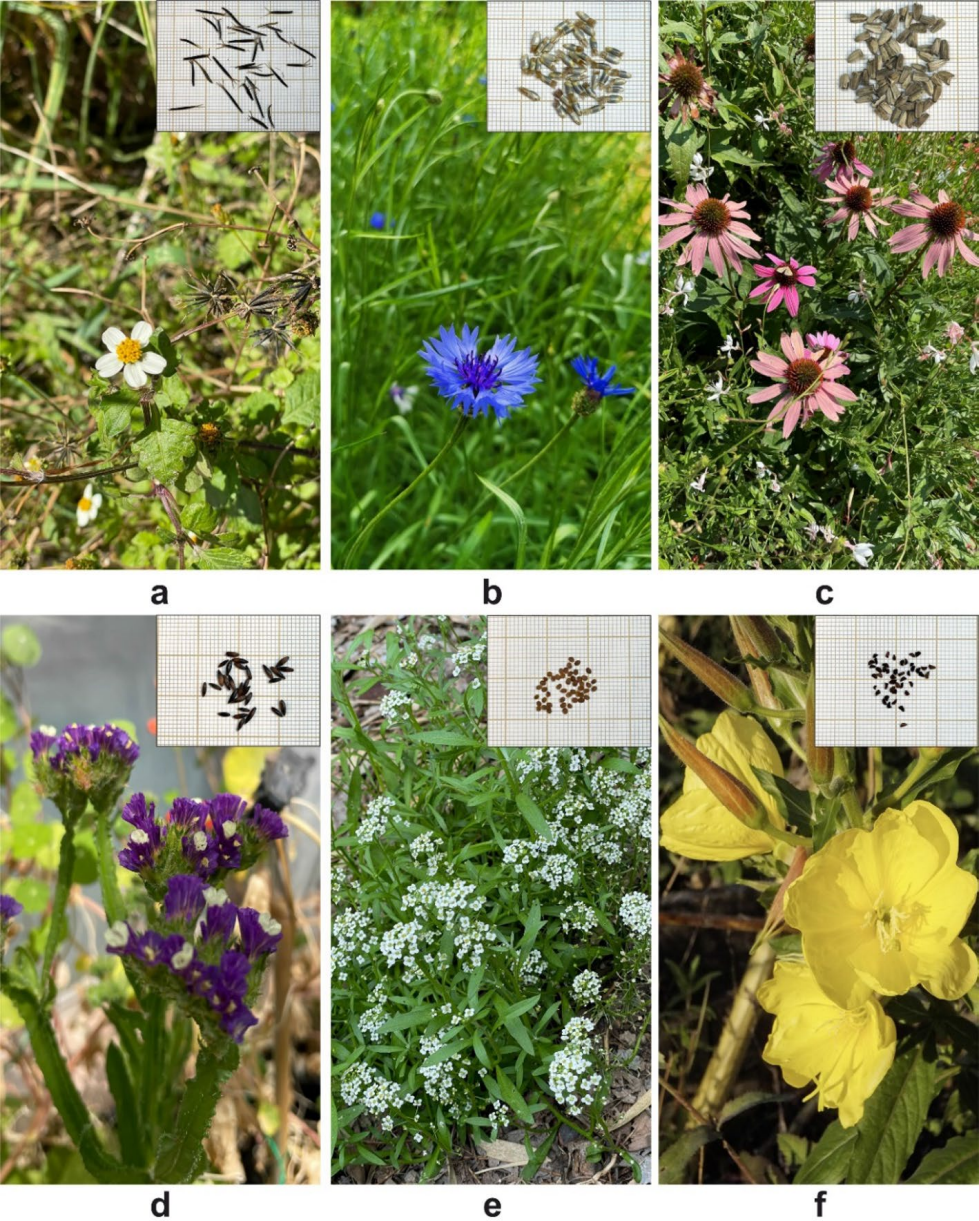
The studied species and their propagules: (**a**) *Bidens pilosa*, (**b**) *Centaurea cyanus*, (**c**) *Echinacea purpurea,* (**d**) *Limonium sinuatum,* (**e**) *Lobularia maritima,* (**f**) *Oenothera biennis.*

In the present study, six ornamental plants (Fig. 5) were included, several of which have very high invasiveness ratings according to the “Invasive Species Compendium”^16,17^. Seeds from commercial sources (CANTUESO Natural Seeds, Córdoba, Spain; Vilmorin Seed Generation, Paris, France) or from wild plants collected in the autumn of 2021 in Valencia, Spain (*B. pilosa*) were used as starting materials for the experimental setup. The study was conducted in the laboratories and greenhouses of the Institute for the Conservation and Improvement of Valencian Agrodiversity (COMAV), Polytechnic University of Valencia, Valencia, Spain.

*Bidens pilosa* L. (Asteraceae) is native to Central and South America but widespread in tropical, subtropical, and temperate regions, where it behaves as an aggressive weed, affecting important crops^29^. The plant has many fruits (cypsela) with 2–4 barbs that adhere to animal fur, bird feathers, or human clothing, increasing the risk of introduction. It generally shows significant demographic trends^30^ invading anthropised settings and natural areas of ecological importance like wetlands and stream beds. Its early onset and fast germination are pre-adaptive traits common in many invasive species^19^.

*Centaurea cyanus* L. (Asteraceae) was a prevalent weed in cereal fields and meadows^31^, probably originating in SE Europe and spreading along trade paths^32^. Farmers regarded it as a nuisance for generations, and some European countries still do^33^, although pesticide use and industrialised agriculture led to its severe populational decline in Western Europe^34^. This now-threatened species in Western Europe is used as an indicator of biodiversity in crop fields^31^. The species is typically found in cultivated areas with cereal crops, wastelands, and roadsides. Its altitudinal distribution ranges from 0 to 1800 m above sea level, and it is drought-tolerant^35^. As a garden escapee, the species became invasive in prairies and grasslands across the US, notably in the Northwest^36^. It has recently been reported as a newcomer in northeast India^37^.

*Echinacea purpurea* L. (Asteraceae) is native to the central to southeastern United States and was introduced in Europe by the end of the seventeenth century. However, its cultivation was intensified in the twentieth century due to its use as a medicinal plant^38^. This plant does not seem to be problematic outside its native territory; a study on commercial nurseries from Estonia catalogued this species as a non-escaping alien^39^, and it was included in the category of species with the lowest invasion risk in an assessment of alien invasive species in the mountains of NW China^40^.

*Limonium sinuatum* (L.) Mill. (Plumbaginaceae) is a recretohalophyte with salt excretory glands, moderately tolerant to salinity and drought resistant^41,42^. In its natural environments, it occurs mainly in rocky and sandy coastal areas^43^. The species is reported to be moderately invasive outside its native range in regions with a Mediterranean climate. In southern California, it has been reported in coastal grasslands, sandy marshes, and along roadsides^44^. In Australia, it is listed as an environmental weed occurring in disturbed sites, waste areas, roadsides, saline wetlands and riparian and mallee woodland communities^45^. Similarly, in South Africa, it invades roadsides and disturbed coastal areas but also penetrates fynbos vegetation and the Karoo^46^.

*Lobularia maritima* L. (Brassicaceae) has a Circum-Mediterranean and Macaronesian distribution. In the Iberian Peninsula, it is found on coastal sandbanks, consolidated dunes, pine forest clearings, scrubland on sandy soils, and stony road slopes^47^. It is a facultative halophyte^48^ and drought-resistant^49^. The species can naturalise in Europe, North America, Asia, Australia, and Oceania^47^. It was introduced in the northern United States around 1856; however, by the late nineteenth century, it was already a naturalised species that escaped from gardens^50^ and is currently recognised as invasive in California^51^. In Australia, it is an environmental weed that grows in disturbed, anthropised coastal areas and a few ecologically important coastal dunes^52^.

*Oenothera biennis* L. (Onagraceae) is a native of N America but recognised as an invasive weed in many continents, including Europe. In Spain, it is common in the country’s north in degraded riparian areas and other nitrogen-rich wet habitats^30^. Although it has been reported as a poor early-stage competitor since it requires an area free of vegetation to establish^20^, *O. biennis* has a high invasive potential. It has been reported to tolerate moderate salinity^53^ and drought^54^. The species invades mostly anthropised associated habitats, so its presence in natural areas such as riparian environments may indicate the degradation of these ecosystems^30^.

### Germination assays

In vitro germination assays were carried out with four replications per treatment and species, with 25 seeds placed in 55 mm-diameter Petri dishes on two layers of filter paper. Beforehand, the seeds were weighed and measured. The paper was moistened with 1.5 mL of distilled water, for the control, or with increasing concentrations of PEG 6000 (Polyethylene Glycol), resulting in osmotic potentials of − 0.25 MPa, − 0.5 MPa, − 0.75 MPa and − 1 MPa. The Van’t Hoff equation^55^ was used to calculate the PEG quantities required to provide the given osmotic potentials. The Petri dishes were incubated in a growth chamber at 25 °C with a 12 h photoperiod within clear zippered bags to avoid evaporation. Every day, the number of germinated seeds, defined as those with a minimum of 1 mm radicle, was counted for 30 days. The germination potential was expressed as a percentage of germination (GP), and the germination rate was determined as mean germination time (MGT) using the formula emergence:

$$MGT = \sum \frac{Dn}{\sum n}$$

where D is the number of days since the start of the germination test and n is the number of seeds that germinated on day D^56^. Furthermore, FGD, first day of germination, LGD, last day of germination (LGD), TSG, time spread of germination (the differences in time between the last germination day and the first germination day) were also calculated.

Other indexes calculated were GI and SE. Germination index (GI), which is a strong predictor of germination success and speed^57^, was derived using the equation:

$$GI =\frac{\sum G}{T}$$

where G is the number of seeds germinating on a particular day, and T is the number of days between the beginning of the experiment and the actual day. Speed of emergence (SE)^58^, used to compute germinative energy via germination speed, is calculated as follows:

$$SE = \left[\frac{number\, of\, germinated\, seeds\, on \,the \,first \,day \,of \,germination}{number \,of\, germinated\, seeds \,on \,the \,last\, day\, of \,germination}\right]\times 100$$

After ten days of germination, the lengths of germinated seeds' radicles and hypocotyls were measured and analysed using Digimizer v.4.6.1 software (MedCalc Software, Ostend, Belgium, 2005–2016) and **s**eedling vigour index (SVI), using the equation described in^59^:

$$SVI =\frac{Seedling length, in mm \times Germination percentage}{100}$$

Recovery treatments were given to seeds that did not germinate after 30 days in the presence of PEG. The seeds were rinsed before being transferred to new Petri dishes with distilled water and incubated for 20 days under the same conditions as in the previous germination tests. In the recovery experiments, germination percentages were estimated according to Zaman et al.^60^.

### Plant growth process and applied water stress treatment

Seedlings from the germination assays mentioned above were transplanted manually into 12 cm diameter plastic pots filled with commercial peat (26% organic carbon, pH 7.0, and EC = 0.6 dS m^−1^), placed in the greenhouse, and irrigated manually twice a week with tap water. Temperatures were between 20 ± 1.6 and 28 ± 2.1 °C, while RH (relative humidity) ranged between 65 ± 8.7 and 93 ± 3.1% during the trials, which took place during a 16-h light cycle. The water stress treatments started five weeks after transplanting the seedlings, when the plantlets were completely formed, using five biological repetitions (individual plants) per species and treatment. The pots were placed in plastic trays (10 pots per tray) and received irrigation twice weekly with tap water supplied to each tray. The control plants received 1.5 L of water per irrigation, the intermediate water stress treatment (IWS) received half of this quantity (0.75 L), and the severe water stress treatment (SWS) received no water at all. When the soil moisture of the SWS plants reached 3–5% after four weeks, the plants were harvested and processed for additional biochemical assays. The aerial parts were removed from the roots, which were cleaned with a brush before measuring and weighing both components (roots and shoots) separately. A sample of the harvested material was weighed both before (fresh weight, FW) and after 72 h of drying at 65 °C (dry weight, DW), and the water content of roots and shoots was determined using the following equation:

$$WC\% = \left[\frac{FW - DW}{FW}\right]\times 100$$

Fresh plant material was frozen in liquid N_2_ and stored at − 75 °C, whereas dry plant material was kept at ambient temperature in tightly sealed paper bags.

**Figure 6:**
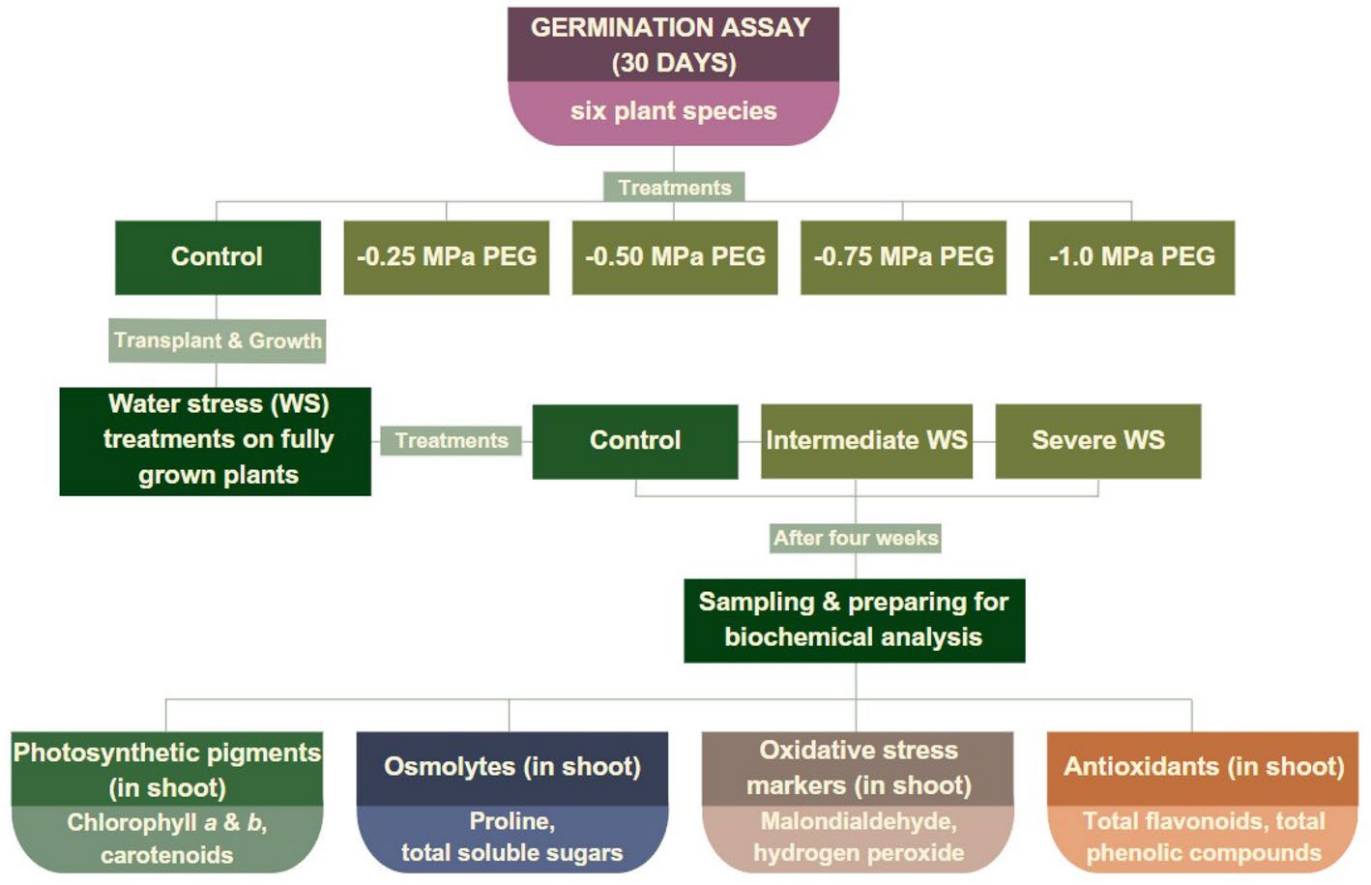
Flowchart of the experimental procedure: stress treatments during seed germination and vegetative plant growth.

Figure 6 synthesizes the experimental procedure in this comparative study from seed germination to biochemical analysis.

### Photosynthetic pigments

Fresh shoot material (50 mg) was ground and extracted in ice-cold 80% acetone overnight. The samples were centrifuged, and the absorbance of the supernatant was then measured at 470 nm, 646 nm, and 663 nm. The contents of chlorophyll *a* (Chl *a*), chlorophyll *b* (Chl *b*), and carotenoids (Caro) were determined using the equations outlined in Lichtenthaler and Wellburn^61^, and expressed in mg g^−1^ DW.

### Quantification of osmolytes

The shoot proline (Pro) concentration was measured as previously published by Bates et al.^62^. Fresh ground shoot material (50 mg) was extracted in 3% (w/v) sulphosalicylic acid; the samples were mixed with acid ninhydrin, incubated in a water bath at 95 °C for 1 h, chilled on ice, and then extracted with toluene. The organic phase's absorbance was measured spectrophotometrically at 520 nm. Samples of known Pro concentration were assayed in parallel to obtain a standard curve, and Pro concentrations were expressed as µmol g^−1^ DW.

Total soluble sugars (TSS) contents were calculated using the method of Dubois et al.^63^. Freshly crushed leaves (50 mg) were extracted overnight with 80% (v/v) methanol. Following centrifugation, 5% phenol and concentrated sulphuric acid were added to the supernatant to cause an exothermic reaction that caramelised the extracted sugars, and the sample absorbance was determined at 490 nm. TSS concentrations were expressed as equivalents of glucose, used as the standard (mg eq. glucose g^−1^ DW).

### Oxidative stress markers and antioxidant compounds determination

Malondialdehyde and hydrogen peroxide are frequently used as biochemical markers of oxidative stress. For MDA quantification, the methanol extracts were combined with 0.5% thiobarbituric acid (TBA) in 20% trichloroacetic acid (TCA), or with 20% TCA without TBA for the controls, and then incubated for 15 min at 95 °C in a water bath. The reactions were stopped on ice, and the samples were centrifuged for 10 min at 4 °C, at 13,300×*g*. Finally, the supernatants' absorbance was measured at 532 nm. MDA concentrations were determined after subtracting non-specific absorbance at 600 and 440 nm, using the equations reported by Hodges et al.^64^, based on the molar extinction coefficient of the MDA-TBA adduct at 532 nm (ε_532_ = 155 mM^−1^ cm^−1^). Finally, MDA concentrations were calculated in nmol g^−1^ DW.

Hydrogen peroxide (H_2_O_2_) was determined as previously described^65^. Fresh leaf material (50 mg) was extracted with a 0.1% (w/v) trichloroacetic acid (TCA) solution, and the extracts were centrifuged at 13,300×*g* for 15 min at 4 °C. A 500 µL supernatant aliquot was combined with 500 µL of 10 mM potassium phosphate buffer (pH 7) and 1 mL of 1 M potassium iodide. The absorbance was measured at 390 nm, and a standard curve was created using samples with known H_2_O_2_ concentrations that were tested in parallel. H_2_O_2_ values were expressed in µmol g^−1^ DW.

Total phenolic compounds (TPC) and total flavonoids (TF) were measured in the same methanol extracts as total soluble sugars (TSS). TPC were determined using the Blainski et al.^66^ method, which involved a reaction with the Folin-Ciocalteu reagent and Na_2_CO_3_; the reaction mixtures were incubated at room temperature for 90 min in the dark, and the absorbance was measured at 765 nm. Gallic acid (GA) was used as the standard, and TPC contents were expressed as mg eq. GA g^−1^ DW.

Total flavonoids (TF) were measured using the method developed by Zhishen et al.^67^, which involves the nitration of aromatic rings containing a catechol group with NaNO_2_, followed by a reaction with AlCl_3_ at basic pH. Finally, the absorbance of the samples was measured at 510 nm, and the TF contents were expressed as equivalents of the standard catechin (mg eq. C g^−1^ DW).

### Statistical analysis

The data were statistically analysed using SPSS Statistics (IBM SPSS Statistics). To analyse the influence of the water stress treatments (CON, IWS, and SWS) on the species, one-way ANOVA was performed independently for each species. The Tukey Honestly Significant Difference (HSD) post hoc test was used to identify statistically significant differences in the mean values of the treatments, with a significance level of p < 0.05. Two-way ANOVA was used to examine the influence of species in addition to the effect of treatment, as well as the significance of the interaction of the two components.

In this study, we conducted separate principal component analyses (PCA) for the germination stage (PCAg) and the vegetative growth stage (PCAv). For each stage, two distinct PCAs were performed: one on control data and the other on data collected under stress conditions. These separate PCAs enabled us to capture the maximum variance and focus on informative features in normal and stress-induced situations. The PCA involved centring and scaling quantitative variables, followed by diagonalisation of the correlation matrix to extract the corresponding eigenvectors and eigenvalues. Correlation coefficients were calculated to assess the relationships between the principal components and the active variables. Variable relevance was determined based on associated p-values.

For the germination phase (PCAg), the following quantitative variables were considered: germination percentage (GP), mean germination time (MGT), first germination day (FGD), last germination day (LGD), total spread of germination (TSG), speed of emergence (SE), germination index (GI), hypocotyl length (HypL), radicle length (RadL), seed length (Sle), seed width (Swi), and seed weight (SW). Under drought stress conditions, seed length (Sle), seed width (Swi), and seed weight (SW) were excluded since their values are inherently unaffected by water stress. The six ornamental species and the four levels of drought stress were treated as supplementary categorical variables, meaning that they were not included in the calculation of the principal components.

For the vegetative growth stage (PCAv), the following quantitative variables were considered: shoot fresh weight (FWs), root fresh weight (FWr), leaf number (Lno), root length (RL), shoot length (SL), root water content (WCr), shoot water content (WCs), chlorophyll *a* (Chl a), chlorophyll *b* (Chl b), carotenoids (Caro), proline (Pro), malondialdehyde (MDA), total flavonoids (TF), total phenolic compounds (TPC), total soluble sugars (TSS), and hydrogen peroxide (H_2_O_2_). To account for species-specific variations and facilitate comparisons, the FWr, FWs, Lno, RL, and SL data were normalised by expressing them as percentage reductions relative to the control values. Additionally, the analysis treated the six ornamental species, and the three levels of drought stress as supplementary categorical variables. The six ornamental species and the three drought stress levels were considered supplementary categorical variables.

Based on the information obtained from the PCA conducted for the germination stage, a correlation analysis was performed to explore the relationships between the germination indices found to be significant in characterizing the different responses to drought stress of the selected species. Pairwise Pearson's correlation coefficients were calculated to assess the associations between these variables, with significance tested at a level of α = 0.05. The PCA and correlation analysis were conducted using RStudio version 4.1.3.

### Declaration on ethics and research permits

All procedures were conducted in accordance with the guidelines. No permissions or licenses were required. The taxonomical identity of plants grown from seeds was checked by M.B. specialist in botany, and voucher specimens were deposited at VALA (the Herbarium of the Polytechnic University of Valencia, Spain), with the deposition numbers: VALA 9635, VALA 9636, VALA 9637, VALA 9638, VALA 9639, VALA 9640).

### Supplementary Information

Supplementary Information.

## Data Availability

Correspondence and requests for materials should be addressed to A.F.S.
