# Supplementary material for: Responses of different invasive and non-invasive ornamental plants to water stress during seed germination and vegetative growth
Source: Sci Rep. 2023 Aug 16;13:13281. doi: 10.1038/s41598-023-40517-7 (PMC10432408; doi:10.1038/s41598-023-40517-7)
Supplement: Supplementary file 1 — Supplementary Information. [file 41598_2023_40517_MOESM1_ESM.docx]

**Table S1:** Parameters of germination with mean values ± SE of four invasive grass species after 30 days of applied osmotic stress and after 20 days of recovery treatment after 20 days. Different lowercase letters within the bars indicate significant differences between treatments within one species, according to Tukey post-hoc test (p < 0.05). Abbreviations: germination percentage reduction (rGP), first germination day (FGD), last germination day (LGD), time spread germination (TSG), speed of emergence (SE), germination index (GI).

| **Para-meter** | **Assay** | **Treat** | ***B. pilosa*** | ***C. cyanus*** | ***E. purpurea*** | ***L. sinuatum*** | ***L. maritima*** | ***O. biennis*** |
| --- | --- | --- | --- | --- | --- | --- | --- | --- |
| **rGP (%)** | **Germination** | -0.25 | -12.0 ± 3.6 | -50.9 ± 4.9 | 4.2 ± 5.1 | 6.8 ± 11.1 | -2.5 ± 1.4 | -2.5 ± 1.0 |
|  |  | -0.5 | -4.8 ± 3.6 | -45 ± 12.1 | 23.8 ± 8.5 | 6.8 ± 2.2 | 12.5 ± 5.2 | 3.8 ± 2.0 |
|  |  | -0.75 | 60.2 ± 4.1 | -21.5 ± 5.0 | 45.7 ± 7.8 | 13.6 ± 8.4 | 87.5 ± 5.9 | 13.3 ± 3.6 |
|  |  | -1 | n.g. | 45 ± 3.2 | n.g. | 84.9 ± 8.4 | n.g. | n.g. |
| **FGD (days)** | **Germination** | C | 1.0 ± 0.0 | 1.0 ± 0.0 | 2.2 ± 0.2 | 1.2 ± 0.2 | 2.0 ± 0.0 | 2.0 ± 0.0 |
|  |  | -0.25 | 2.0 ± 0.0 | 2.0 ± 0.0 | 2.7 ± 0.2 | 1.0 ± 0.0 | 2.0 ± 0.0 | 2.0 ± 0.0 |
|  |  | -0.5 | 2.2 ± 0.2 | 2.0 ± 0.0 | 4.5 ± 0.5 | 1.7 ± 0.2 | 3.0 ± 0.0 | 3.0 ± 0.0 |
|  |  | -0.75 | 9.5 ± 1.8 | 2.0 ± 0.0 | 7.2 ± 0.2 | 4.2 ± 0.2 | 6.7 ± 2.8 | 5.0 ± 0.5 |
|  |  | -1 | n.g. | 3.0 ± 0.0 | n.g. | 4.2 ± 1.7 | n.g. | n.g. |
|  | **Recovery** | -0.25 | - | - | 1.0 ± 0.0 | - | - | - |
|  |  | -0.5 | 2.0 ± 1.0 | 5.3 ± 2.6 | 1.0 ± 0.0 | 1.0 ± 0.0 | - | 0.3 ± 0.3 |
|  |  | -0.75 | 1.0 ± 0.0 | 1.6 ± 0.6 | 1.0 ± 0.0 | 1.0 ± 0.0 | 1.0 ± 0.0 | 1.3 ± 0.3 |
|  |  | -1 | 1.0 ± 0.0 | 0.6 ± 0.6 | 1.0 ± 0.0 | 1.0 ± 0.0 | 1.0 ± 0.0 | 1.2 ± 0.2 |
| **LGD (days)** | **Germination** | C | 5.5 ± 1.7 | 2.5 ± 0.2 | 9.7 ± 0.4 | 11.2 ± 4.2 | 8.7 ± 0.9 | 4.7 ± 0.2 |
|  |  | -0.25 | 9.0 ± 0.0 | 10.7 ± 3.6 | 14.2 ± 4.1 | 14.0 ± 3.7 | 9.7 ± 0.4 | 6.2 ± 1.0 |
|  |  | -0.5 | 16.7 ± 0.9 | 7.0 ± 0.0 | 14.7 ± 2.7 | 13.7 ± 2.3 | 17.2 ± 0.4 | 10.0 ± 2.6 |
|  |  | -0.75 | 18.0 ± 0.0 | 13.7 ± 2.2 | 19.2 ± 2.5 | 13.2 ± 3.9 | 9.5 ± 3.3 | 16.7 ± 2.0 |
|  |  | -1 | n.g. | 15.7 ± 0.4 | n.g. | 9.7 ± 3.8 | n.g. | n.g. |
|  | **Recovery** | -0.25 | - | - | 1 ± 0 | - | - | - |
|  |  | -0.5 | 2.0 ± 1.0 | 5.3 ± 2.6 | 3.3 ± 0.3 | 1.0 ± 0.0 | - | 0.3 ± 0.3 |
|  |  | -0.75 | 5.3 ± 4.3 | 1.6 ± 0.6 | 3.0 ± 0.0 | 2.3 ± 1.3 | 1.0 ± 0.0 | 3.3 ± 0.6 |
|  |  | -1 | 1.0 ± 0.0 | 0.6 ± 0.6 | 3.2 ± 0.4 | 1.0 ± 0.0 | 1.6 ± 0.3 | 3.0 ± 0.0 |
| **TSG (days)** | **Germination** | C | 4.5 ± 1.7 | 1.5 ± 0.2 | 7.5 ± 0.6 | 10.2 ± 4.2 | 6.7 ± 0.9 | 2.7 ± 0.2 |
|  |  | -0.25 | 7.0 ± 0.0 | 8.7 ± 3.6 | 11.5 ± 4 | 13.0 ± 3.7 | 7.7 ± 0.4 | 4.2 ± 1.0 |
|  |  | -0.5 | 14.5 ± 0.9 | 5.0 ± 0.0 | 10.2 ± 2.7 | 12.0 ± 2.4 | 14.2 ± 0.4 | 7.0 ± 2.6 |
|  |  | -0.75 | 8.5 ± 1.8 | 11.7 ± 2.2 | 12.0 ± 2.4 | 9.0 ± 3.7 | 3.0 ± 1.7 | 11.7 ± 2.4 |
|  |  | -1 | n.g. | 12.7 ± 0.4 | n.g. | 5.7 ± 3.1 | n.g. | n.g. |
|  | **Recovery** | -0.25 | - | - | 1.0 ± 0.0 | - | - | - |
|  |  | -0.5 | 0.6 ± 0.3 | 0.6 ± 0.3 | 2.3 ± 0.3 | 1.0 ± 0.0 | - | 0.3 ± 0.3 |
|  |  | -0.75 | 5.0 ± 4.0 | 1.0 ± 0.0 | 3.0 ± 0.0 | 2.0 ± 1.0 | 1.0 ± 0.0 | 2.0 ± 0.5 |
|  |  | -1 | 1.0 ± 0.0 | 0.3 ± 0.3 | 2.2 ± 0.4 | 1.0 ± 0.0 | 1.0 ± 0.0 | 1.7 ± 0.2 |
| **SE** | **Germination** | C | 12.0 ± 3.1 | 67.2 ± 12.1 | 16.1 ± 7.8 | 21.6 ± 11.9 | 64.4 ± 5.9 | 76.4 ± 7.4 |
|  |  | -0.25 | 31.9 ± 5.8 | 72.7 ± 6.1 | 30.0 ± 8.7 | 7.9 ± 2.2 | 22.0 ± 1.7 | 11.2 ± 2.5 |
|  |  | -0.5 | 5.8 ± 1.3 | 52.6 ± 8.2 | 15.4 ± 3.9 | 8.7 ± 1.5 | 34.5 ± 3.9 | 17.5 ± 2.4 |
|  |  | -0.75 | 15.0 ± 2.0 | 11.0 ± 2.8 | 17.9 ± 5.4 | 15.1 ± 5.3 | 53.7 ± 21.7 | 19.8 ± 8.7 |
|  |  | -1 | n.g. | 18.0 ± 3.6 | n.g. | 36.9 ± 22.1 | n.g. | n.g. |
|  | **Recovery** | -0.25 | - | - | 100.0 ± 0.0 | - | - | - |
|  |  | -0.5 | 66.6 ± 33.3 | 66.6 ± 33.3 | 33.3 ± 9.6 | 100.0 ± 0.0 | - | 33.3 ± 33.3 |
|  |  | -0.75 | 97.6 ± 2.3 | 100.0 ± 0.0 | 42.8 ± 7.3 | 95.8 ± 4.1 | 100.0 ± 0.0 | 53.3 ± 14.8 |
|  |  | -1 | 100.0 ± 0.0 | 33.3 ± 33.3 | 77.3 ± 3.8 | 100.0 ± 0.0 | 85.1 ± 9.7 | 47.0 ± 10.6 |
| **GI** | **Germination** | C | 10.9 ± 0.8 | 10.2 ± 0.6 | 6.2 ± 0.1 | 8.4 ± 1.3 | 8.1 ± 0.4 | 14.8 ± 0.6 |
|  |  | -0.25 | 8.2 ± 0.5 | 8.2 ± 0.3 | 5.1 ± 0.2 | 6.7 ± 0.8 | 6.1 ± 0.2 | 7.0 ± 0.2 |
|  |  | -0.5 | 4.4 ± 0.1 | 7.2 ± 0.8 | 2.3 ± 0.2 | 4.1 ± 0.4 | 3.5 ± 0.2 | 5.4 ± 0.1 |
|  |  | -0.75 | 0.6 ± 0.1 | 3.9 ± 0.4 | 1.2 ± 0.2 | 2.4 ± 0.3 | 0.3 ± 0.1 | 2.7 ± 0.1 |
|  |  | -1 | n.g. | 1.2 ± 0.1 | n.g. | 0.3 ± 0.2 | n.g. | n.g. |
|  | **Recovery** | -0.25 | - | - | 1 ± 0 | - | - | - |
|  |  | -0.5 | 0.2 ± 0.1 | 0.1 ± 0.0 | 3.5 ± 0.3 | 7.3 ± 0.3 | - | 0.3 ± 0.3 |
|  |  | -0.75 | 12.6 ± 0.3 | 0.7 ± 0.2 | 5.6 ± 0.6 | 7.7 ± 0.2 | 7.6 ± 0.3 | 2.6 ± 0.6 |
|  |  | -1 | 24.6 ± 0.3 | 0.3 ± 0.3 | 17 ± 0.4 | 20.0 ± 0.0 | 9.0 ± 0.0 | 13.6 ± 1.3 |

**Table S2:** Two-way ANOVA (F-values) considering the effect of Species (S), Treatment (T), and their interactions (S × T) on seed germination parameters: germination percentage (GP), mean germination time (MGT), first germination day (FGD), last germination day (LGD), time spread germination (TSG), speed of emergence (SE), germination index (GI), hypocotyl length (HypL), hypocotyl length reduction (rHypL), radicle length (RadL), radicle length reduction (rRad).

| **Parameter** | **S** | **T** | **S × T** |
| --- | --- | --- | --- |
| **GP** | 6.499 *** | 322.718 *** | 14.570 *** |
| **MGT** | 1.744 * | 24.065 *** | 3.816 *** |
| **FGD** | 1.018 ^ns^ | 10.534 *** | 1.665 ^ns^ |
| **LGD** | 3.424 *** | 24.935 *** | 4.894 *** |
| **TSG** | 3.911 *** | 16.995 *** | 6.044 *** |
| **SE** | 3.728 *** | 11.389 *** | 4.658 *** |
| **GI** | 5.189 *** | 209.037 *** | 4.059 *** |
| **SVI** | 29.257 *** | 378.237 *** | 23.892 *** |
| **HypL** | 40.349 *** | 418.968 *** | 21.053 *** |
| **rHypL** | 5.477 *** | 699.368 *** | 10.863 *** |
| **RadL** | 52.092 *** | 419.819 *** | 29.706 *** |
| **rRadL** | 7.191 *** | 413.470 *** | 9.958 *** |

*, **, *** significant at p = 0.05, 0.01 and 0.001 respectively; ns: not significant

**Table S3:** Two-way ANOVA (F-values) considering the effect of Species (S), Treatment (T), and their interactions (S × T) on growth parameters: leaf number (Lno), leaf number reduction (rLno), root length (RL0, root length reduction (rRL), shoot length (SL), shoot length reduction (rSL), root fresh weight (FWr), root fresh weight reduction (rFWr), shoot fresh weight (FWs), shoot fresh weight reduction (rFWs), water content root (WCr), water content shoot (WCs).

| **Parameter** | **S** | **T** | **S × T** |
| --- | --- | --- | --- |
| **Lno** | 136.585 *** | 8.587 *** | 1.461 ^ns^ |
| **rLno** | 2.831 * | 22.329 *** | 3.288 *** |
| **RL** | 15.581 *** | 5.060 ** | 1.247 ^ns^ |
| **rRL** | 4.061 ** | 6.013 ** | 1.163 ^ns^ |
| **SL** | 112.222 *** | 26.710 *** | 3.301 *** |
| **rSL** | 0.775 ^ns^ | 22.419 *** | 2.907 ** |
| **FWr** | 25.681 *** | 42.213 *** | 3.782 *** |
| **rFWr** | 1.902 ^ns^ | 27.492 *** | 1.177 ^ns^ |
| **FWs** | 19.163 *** | 63.579 *** | 1.725 * |
| **rFWs** | 0.253 ^ns^ | 91.800 *** | 0.548 ^ns^ |
| **WCr** | 6.303 *** | 54.787 *** | 10.676 *** |
| **WCs** | 1.608 ^ns^ | 24.352 *** | 0.621 ^ns^ |

*, **, *** significant at p = 0.05, 0.01 and 0.001 respectively; ns: not significant

**Table S4:** Two-way ANOVA (F-values) considering the effect of Species (S), Treatment (T), and their interactions (S × T) on biochemical parameters: chlorophyll *a* (Chl A), chlorophyll *b* (Chl B), carotenoids (Caro), proline (Pro), total soluble sugars (TSS), malondialdehyde (MDA), hydrogen peroxide (H_2_O_2_), total flavonoids (TF), total phenolic compounds (TPC).

| **Parameter** | **S** | **T** | **S × T** |
| --- | --- | --- | --- |
| **Chl A** | 3.094 * | 8.884 *** | 1.258 ^ns^ |
| **Chl B** | 2.502 * | 9.140 *** | 0.974 ^ns^ |
| **Caro** | 7.765 *** | 7.643 *** | 1.629 ^ns^ |
| **Pro** | 26.719 *** | 140.137 *** | 14.201*** |
| **TSS** | 20.211 *** | 0.076 ^ns^ | 1.174 ^ns^ |
| **MDA** | 14.853 *** | 4.818 * | 1.001 ^ns^ |
| **H_2_O_2_** | 11.865 *** | 2.078 ^ns^ | 4.772 *** |
| **TF** | 9.358 *** | 0.313 ^ns^ | 1.241 ^ns^ |
| **TPC** | 5.190 *** | 0.519 ^ns^ | 1.259 ^ns^ |

*, **, *** significant at p = 0.05, 0.01 and 0.001 respectively; ns: not significant


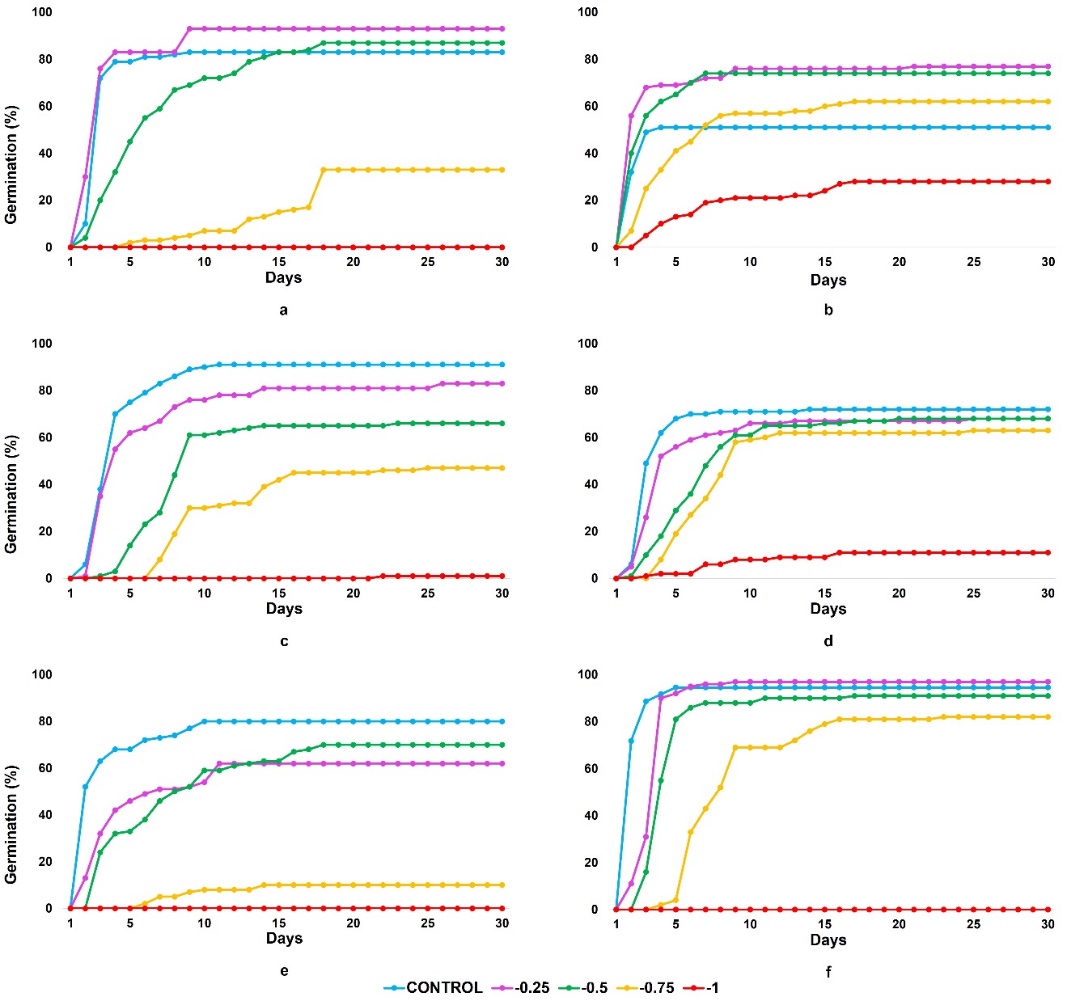


**Figure S1.** Evolution of seed germination (%) over 30 days of osmotic stress treatments using PEG at the indicated concentrations, expressed as osmotic potential (MPa) in (a) *Bidens pilosa*, (b) *Centaurea cyanus*, (c) *Echinacea purpurea,* (d) *Limonium sinuatum,* (e) *Lobularia maritima,* (f) *Oenothera biennis.* Means of cumulative germination percentages of four technical replicas (plates) are presented for each treatment and species. Control: germination in distilled water.
